# Advanced Microparticulate/Nanoparticulate Respirable Dry Powders of a Selective RhoA/Rho Kinase (Rock) Inhibitor for Targeted Pulmonary Inhalation Aerosol Delivery

**DOI:** 10.3390/pharmaceutics13122188

**Published:** 2021-12-17

**Authors:** Priya Muralidharan, Don Hayes, Jeffrey R. Fineman, Stephen M. Black, Heidi M. Mansour

**Affiliations:** 1 Department of Pharmaceutical Sciences, College of Pharmacy, The University of Arizona, Tucson, AZ 85721, USA; muralidharan@pharmacy.arizona.edu; 2 Departments of Pediatrics and Internal Medicine, Lung and Heart-Lung Transplant Programs, Nationwide Children’s Hospital, The Ohio State University College of Medicine, Columbus, OH 43205, USA; don.hayes@cchmc.org; 3 UCSF School of Medicine & Benioff Children’s Hospital, San Francisco, CA 94158, USA; Jeff.Fineman@ucsf.edu; 4 Center for Translational Science, Florida International University, Port Saint Lucie, FL 34987, USA; stblack@fiu.edu; 5 The BIO5 Research Institute, The University of Arizona, Tucson, AZ 85721, USA; 6 Institute of the Environment, The University of Arizona, Tucson, AZ 85721, USA

**Keywords:** particle engineering design, solid-state characterization, pulmonary hypertension, targeted lung delivery, advanced spray drying, in vitro human lung cells, in vitro TEER, air-interface culture (AIC), dry powder inhaler

## Abstract

Pulmonary hypertension (PH) is a progressive disease that eventually leads to heart failure and potentially death for some patients. There are many unique advantages to treating pulmonary diseases directly and non-invasively by inhalation aerosols and dry powder inhalers (DPIs) possess additional unique advantages. There continues to be significant unmet medical needs in the effective treatment of PH that target the underlying mechanisms. To date, there is no FDA-approved DPI indicated for the treatment of PH. Fasudil is a novel RhoA/Rho kinase (ROCK) inhibitor that has shown great potential in effectively treating pulmonary hypertension. This systematic study is the first to report on the design and development of DPI formulations comprised of respirable nanoparticles/microparticles using particle engineering design by advanced spray drying. In addition, comprehensive physicochemical characterization, in vitro aerosol aerosol dispersion performance with different types of human DPI devices, in vitro cell-drug dose response cell viability of different human respiratory cells from distinct lung regions, and in vitro transepithelial electrical resistance (TEER) as air-interface culture (AIC) demonstrated that these innovative DPI fasudil formulations are safe on human lung cells and have high aerosol dispersion performance properties.

## 1. Background

Pulmonary hypertension (PH) is a progressive disease that leads to increased intra-arterial pressure in the pulmonary vasculature leading to heart failure. PH is categorized into several types based on its etiology [1]. The cause of PH is not well understood; however, clinical manifestations include pulmonary vasoconstriction, proliferation of smooth muscle cells, migration of inflammatory cells and pulmonary vascular remodeling [2,3,4]. PH is also a serious pulmonary disease that exists concomitantly with other serious chronic pulmonary diseases, such as idiopathic pulmonary fibrosis [5], cystic fibrosis [6], and chronic obstructive lung disease (COPD) [7].

Rho-associated protein kinase, also known as Rho-kinase or ROCK, an enzyme that belongs to the kinase family of serine-threonine kinases AGC, is a downstream effector of Rho GTPase [8,9]. There are two isoforms, ROCK-1 and ROCK-2, which are expressed in multiple tissues, although ROCK 1 is predominantly found in the liver, lung and testis. Rho-kinase has several functions including regulation of cellular contraction, motility, morphology, polarity, cell division and gene expression [8,9]. The Rho kinase inhibitor, Fasudil competitively binds to the ATP binding site in Rho kinase and regulates the phosphorylation of myosin light chain leading to the vasodilation of constricted vessels [3]. Its metabolite, hydroxyfasudil, is also biologically active. Its effect in animal model has shown its role in vascular remodeling [2,3]. Fasudil is an approved drug in Japan for cerebral vasospasm. The Rho/ROCK signaling pathway has been implicated in many lung diseases. Fasudil has been shown to strongly activate the Nrf2 antioxidant pathway [10]. Previously, a reduction in pulmonary arterial pressure also decreased the progression of COPD with Liu et al. studying the effect of Fasudil on a selected number of COPD patients with PH [11]. It was found that the pulmonary artery pressure was significantly reduced in Fasudil-treated patients compared to a control group. An increase in the number of endothelial progenitor cells, which can play a key role in repairing damaged pulmonary arterial endothelium to reduce pulmonary artery pressure, was discovered. Thus, Fasudil reduced the damage and improved reconstruction of pulmonary vascular endothelium [11]. The involvement of Rho/ROCK signaling in the ozone induced airway hyper-responsiveness and inflammation has also been reported [12,13]. Moreover, Fasudil has been shown to decrease both the number of inflammatory cells and the inflammation index, in lung [14]. Fasudil also decreased allergen-induced mucus hypersecretion by down regulating NF-κB and STAT6 [14]. With Rho/ROCK signaling involvement in hypoxia-induced pulmonary fibrosis, Fasudil blocked the development of the fibrosis in another study [15]. Finally, the inhibition of Rho kinase with Fasudil reduced the induction of inflammatory mediators and attenuated septic lung injury [16]. Recently, a prodrug of Fasudil has been reported [17]. Based on the growing evidence in the medical literature, Fasudil appears to be a potential option for the treatment of PH, with it being the only Rho/ROCK inhibitor currently in clinical trials.

Nanopharmaceutical products have greatly improved therapeutics and enabled the treatment of challenging complex diseases [18]. There are many advantages of inhalation aerosols and inhalable nanoparticles [19,20] for targeted pulmonary delivery of therapeutics for the treatment and management of pulmonary diseases [21,22]. Dry powder inhalers (DPIs) have several additional unique advantages over liquid inhalation aerosols [23]. Some key advantages are greater physical and chemical stability of powders in contrast to liquids, solid-state particle engineering of solid particles, a variety of small hand-held DPI devices available for patient use, greater portability of DPIs over nebulizers, and absence of propellants compared to pressurized metered dose inhaler. There have been a few successful studies reported on formulating Fasudil for pulmonary delivery for liquid inhalation aerosol delivery, including liposomes for local delivery and sustained release [24,25], peptide-micelle [26], and surface modified liposomes with homing peptide to target the pulmonary vasculature [27], magnetic liposomes for preferential accumulation [28], and cell based nanoerythrocyte formulations [29].

To the Authors’ knowledge, this is the first study to report on solid-state respirable Fasudil nanoparticles/microparticles as DPIs and with comprehensive physicochemical characterization and in vitro cell studies on human pulmonary cells following successful particle engineering design by advanced organic solution spray drying in closed-mode. The solid-state nanoparticles/microparticles were rationally designed for targeted pulmonary delivery to the deep lung region and smaller airways. In addition, the solid-state properties were tailored and optimized for delivery as DPIs. This study is the first to report on the interactions of these inhalable Fasudil dry powder nanoparticulate/microparticulate formulations with 3 different DPI devices with varying shear stress. The three unit-dose capsule-based devices used in the study, the Aerolizer^®^ (Merck, Kenilworth, NJ, USA), Neohaler^®^ (Novartis, Basel, Switzerland) and Handihaler^®^ (Boehringer Ingelheim, Germany), are FDA -approved human DPI devices. The three distinct devices possess varying shear stress properties that influences the aerosol dispersion. Currently, these devices are used with physical mixtures of drug with large non-inhalable lactose monohydrate carrier particles. However, one of the innovative aspects of this study is that it aims at developing engineered carrier-free DPI formulations of Fasudil. In addition, we report on in vitro interactions of Fasudil with three human pulmonary cell types from different lung regions through in vitro cell viability and in vitro transepithelial electrical resistance (TEER) under air-interface culture (AIC) conditions.

## 2. Experimental: Materials and Methods

### 2.1. Materials

**Figure 1:**
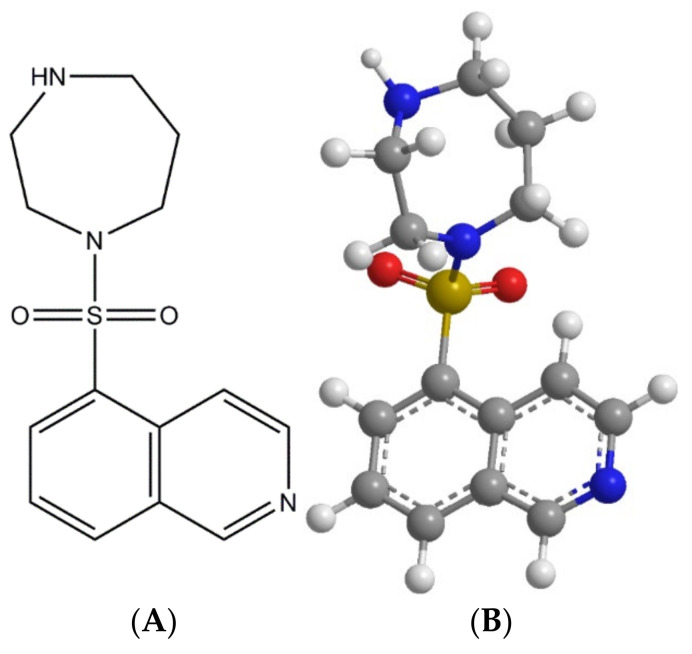
Structures of Fasudil (ChemDraw^®^ Ultra Ver. 10.0.; CambridgeSoft, Cambridge, MA, USA): (**A**). Chemical structure; and (**B**). ball-n-stick 3-D model.

Fasudil (FAS) monohydrochloride hemihydrate salt (FAS) [C_14_H_17_N_3_O_2_S; molecular weight (MW): 291.36 g/mol], shown in Figure 1 (ChemDraw^®^ Ultra Ver. 10.0.; CambridgeSoft, Cambridge, MA, USA), was obtained from LC laboratories (Woburn, MA, USA). Methanol (HPLC grade, ACS-certified grade, purity 99.9%) was obtained from Fisher Scientific (Fair Lawn, NJ, USA). Hydranal^®^ -Coulomat AD was obtained from Sigma–Aldrich (St.Louis, MO, USA). The nitrogen gas used was ultra-high purity (UHP) nitrogen gas (Cryogenics and Gas Facility, The University of Arizona, Tucson, AZ, USA). Raw FAS was stored in sealed glass desiccators over Indicating Drierite™ (Merck KgaA, Darmastadt, Germany) desiccant at temperature below −20 °C. Other chemicals were stored under room conditions.

### 2.2. Methods

Preparation of Spray Dried particles by Organic Solution Advanced Spray Drying in Closed Mode.

An organic solution advanced spray drying process in the absence of water was performed in closed-mode using a Büchi B-290 Mini Spray Dryer (Büchi Labortechnik AG, Flawil, Switzerland) with the Büchi B-295 inert loop (Büchi Labortechnik AG, Flawil, Switzerland) and a high performance cyclone using ultra-high purity (UHP) nitrogen atomizing gas (Cryogenics and gas facility, The University of Arizona, Tucson, AZ, USA) similar to previous studies [30,31]. The feed solution was prepared by dissolving 0.5% *w*/*v* of the drug in methanol. Table 1 lists the spray drying conditions for FAS powders. The stainless steel two-fluid nozzle tip diameter was 0.7 mm with 1.5 mm gas cap. All spray dried (SD) powders were stored in sealed glass desiccators at −20 °C under ambient pressure.

### 2.3. Scanning Electron Microscopy (SEM) and Energy Dispersive X-ray (EDX) Spectrometry

Using conditions similar to those previously reported [30,31,32,33], visual particle size, shape and morphology were imaged using scanning electron microscopy (SEM). The powders were gold coated prior to imaging using 20 µA Argon plasma for 90 s. The electron beam with an accelerating voltage of 30 kV was used at a working distance of 10–10.4 mm. EDX was performed using ThermoNoran systems Six (Thermo Scientific, Waltham, MA, USA) by adjusting spot size until a dead time of 20–30 was obtained.

### 2.4. Particle Sizing and Size Distribution by Image Analysis of SEM Micrographs

The mean size, standard deviation and size range of the particles were determined digitally using Sigma Scan Pro 5.0.0 (Systat, San Jose, CA, USA), using similar conditions that have been previously reported [31,34,35]. Representative micrographs for each particle sample at 15,000× magnification was analyzed by measuring the diameter of at least 100 particles per sample.

### 2.5. X-ray Powder Diffraction (XRPD)

Using conditions similar to those previously reported [30,31,32,33], X-ray powder diffraction (XRPD) patterns of samples were collected at room temperature using PANalytical X’pert diffractometer (PANalytical Inc., Westborough, MA, USA) with a Cu Kα radiation (45 kV, 40 mA, and λ = 1.5406 Å) between 5.0° and 50.0° (2θ) with a scan rate of 2.00°/minute at ambient temperature. The powder samples were filled into glass capillary that was placed on a zero background silicon wafer sample holder and the diffraction pattern was measured with an X’celerator detector.

### 2.6. Differential Scanning Calorimetry (DSC)

Using conditions similar to those previously reported [30,31,32,33], thermal analysis and phase transition measurements were performed with TA Q1000 (TA instruments, New Castle, DE, USA). Approximately 1–10 mg sample was placed into TZero^®^ (New Castle, DE, USA) DSC pans that were hermetically sealed. An empty hermetically sealed aluminum pan was used as a reference pan for all the experiments. The samples were heated from 0.00 °C to 250.00 °C at a scanning rate of 5.00 °C/min.

### 2.7. Hot Stage Microscopy (HSM) under Cross-Polarizers

Using conditions similar to those previously reported [30,31,32,33], thermal activity of the sample was recorded using a Leica DMLP cross-polarizer (Wetzlar, Germany) equipped with a Mettler FP 80 central processor heating unit and Mettler FP82 hot stage (Columbus, OH, USA). Powder was mounted on glass slide and heated from 25.0 °C to 250.0 °C at a heating rate of 5.00 °C/min. The images were digitally captured under 10× optical objective and 10× digital zoom.

### 2.8. Karl Fisher Titration (KFT)

Using conditions similar to previously reported [30,31,32,33], the residual water content of all SD powders were quantified analytically by Karl Fischer titration (KFT) coulometrically with Titroline 7500 trace titrator (SI analytics, Weilheim, Germany). Approximately 2–10 mg of powder was added to the titration cell containing Hydranal^®^ (Charlotte, NC, USA) Coulomat AD reagent. The residual water content was measured after the titration completed.

### 2.9. Confocal Raman Microspectroscopy (CRM), and Chemical Imaging

Confocal Raman microspectroscopy (CRM) was obtained using laser excitation 785 nm with a Renishaw InVia Reflex (Gloucestershire, UK) at the surface using a 20× magnification objective on a Leica DM2700 optical microscope (Wetzlar, Germany) and equipped with a Renishaw inVia Raman system (Gloucestershire, UK). This Renishaw system has a 2400 L/mm grating, with a slit width of 65 μm and a thermoelectrically cooled Master Renishaw CCD detector.

### 2.10. Attenuated Total Reflectance (ATR)-Fourier-Transform Infrared (FTIR) Spectroscopy

ATR-FTIR spectra were obtained using Nicolet Avatar 360 FTIR spectrometer (Varian Inc., Palo Alto, CA, USA) equipped with DTGS detector and a Harrick MNP-Pro (Pleasantville, NY, USA) similar to previous studies [30,31,32,33]. Each spectrum was collected for 32 scans at a spectral resolution of 8 cm^−1^ over the wavenumber range of 4000–400 cm^−1^. A background spectrum was carried out under the same experimental conditions and was subtracted from each sample spectrum.

### 2.11. In Vitro Aerosol Dispersion Performance 

In vitro aerosol dispersion performance was conducted in accordance to USP Chapter <601> specifications [36] using conditions similar to those previously reported [30,31,32,33], the aerosol dispersion performance of spray dried particles was tested using the Next Generation Impactor™ (NGI™) (MSP Corporation, Shoreview, MN, USA) connected to a stainless steel induction port (USP throat) and a mouthpiece adaptor. The flow rate (Q) of 60 L/min using a Copley DFM 2000 digital flow meter (Copley Scientific, Nottingham, UK) and high-capacity oil-free vacuum pump (Copley Scientific, Nottingham, UK). Three FDA-approved human DPI devices Aerolizer^®^ (Merck, Kenilworth, NJ, USA), Neohaler^®^ (Novartis, Basel, Switzerland) and HandiHaler^®^ (Boehringer Ingelheim, Germany) were used and these are all unit-dose capsule-based DPI devices. Quali-V-I clear HPMC size 3 inhalation grade capsules (Qualicaps, Whitsett, NC, USA) were filled with about 10 mg of powder. The aerosol dispersion performance was measured gravimetrically using NGI gravimetric collection cups (MSP Corporation, Shoreview, MN, USA) containing type A/E glass fiber filters with diameter 75 mm (Advantec, Japan) and diameter 55 mm (PALL Corporation, Port Washington, NY, USA), similar to our previous reports (30–33). Three capsules were used per experiment. All experiments were triplicated (*n* = 3) at ambient temperature and humidity. The *ED* (%) Equation (1) was used to express the percentage of *ED* based on the total dose (*TD*) used. The fine particle dose (*FPD*) was defined as the dose deposited on Stages 2 to 7. The fine particle fraction (*FPF*%) Equation (2) was expressed as the percentage of *FPD* to *ED*. The respirable fraction (*RF*%) Equation (3) was used as the percentage of *FPD* to total deposited dose (*DD*) on all impactor stages.

(1) $$Emitted\;Dose\;Fraction\;\left(ED\%\right)=\frac{ED}{TD}\times 100\%$$

(2) $$Fine\;Particle\;Fraction\;\left(FPF\%\right)=\frac{FPD}{ED}\times 100\%$$

(3) $$Respirable\;Fraction\;\left(RF\%\right)=\frac{FPD}{DD}\times 100\%$$

### 2.12. In Vitro Cell Viability

The effect of the drug formulations on the human pulmonary cell lines A549, a human alveolar epithelial lung adenocarcinoma, and H358, an alveolar type II pneumocyte were studied as described [34,37,38]. Both cell lines were grown in Dulbecco’s modified Eagle’s medium (DMEM) advanced 1×, 10% (*v*/*v*) growth medium including fetal bovine serum (FBS), Pen-Strep (100 U/mL penicillin, 100 µg/mL), Fungizone (0.5 µg/mL amphotericin B, 0.41 µg/mL sodium deoxycholate), and 2 mM L-Glutamine in a humidified incubator at 37 °C and 5% CO_2_. The cells were exposed to 100 µL of FAS dissolved in media at different concentrations then incubated for 72 h. A volume of 20 µL of 10 µM resazurin sodium salt was added to each well and the fluorescence intensity was detected after 4 h at 544 nm (excitation) and 590 nm (emission). The relative viability of each sample was calculated as follow:

(4) $$Relative\;Viability\;\left(\%\right)=\frac{Sample\;fluorescence\;intensity\;}{control\;fluorescence\;intensity}\times 100\%$$

### 2.13. In Vitro Transepithelial Electrical Resistance (TEER)

Calu-3 cells, a human lung epithelial cell line, were grown in Eagle’s minimum essential medium (EMEM) growth media with 10% (*v*/*v*) fetal bovine serum (FBS), Pen-Strep (100 U/mL penicillin, 100 µg/mL), Fungizone (0.5 µg/mL amphotericin B, 0.41 µg/mL sodium deoxycholate) as previously reported [34,37]. The cell seeding and growth were performed similar to previous conditions [34,38]. After 10 days of growth, when the cells reached a TEER value of ~1000 Ω/cm^2^ air-interface culture (AIC) conditions were induced by removing media from the apical side and adding 800 μL of media to the basolateral side of the Transwell. The TEER value was measured with an Endohom 12 mm Culture Cup (World precision instruments, Sarasota, FL, USA) where 0.5 mL of media was added to the apical side only for the measurements after which the cells returned to AIC. Once the TEER values reached 500 Ω/cm^2^ in AIC conditions, the cells were exposed to 100 µM of raw Fasudil and SD FAS formulations, delivered using the MicroSprayer^®^ Aerolizer (Penn-Century Inc, Wyndmoor, PA, USA), dissolved in non-supplemented EMEM media. TEER values were then recorded for up to 7 days after aerosol treatment, as previously reported [34,37,38].

### 2.14. Statistical Analysis

The Design of experiments (DoEs) for in vitro aerosol performance was conducted using Design Expert^®^ 8.0.7.1 software (Stat-Ease Corporation, Minneapolis, MN, USA). A full factorial multi-level design was used for in vitro aerosol performance experiment randomization and post-run analysis. Interaction of process parameter on the performance of the SD formulations was evaluated with analysis power of 99.1 and 93 for device resistance and pump rate using Design Expert^®^. All experiments were performed in triplicate (*n* = 3) unless otherwise mentioned, such as the in vitro cell viability (*n* = 6). Results are expressed as mean ± standard deviation.

## 3. Results

### 3.1. Scanning Electron Microscopy (SEM) and Energy Dispersive X-ray (EDX)

**Figure 2:**
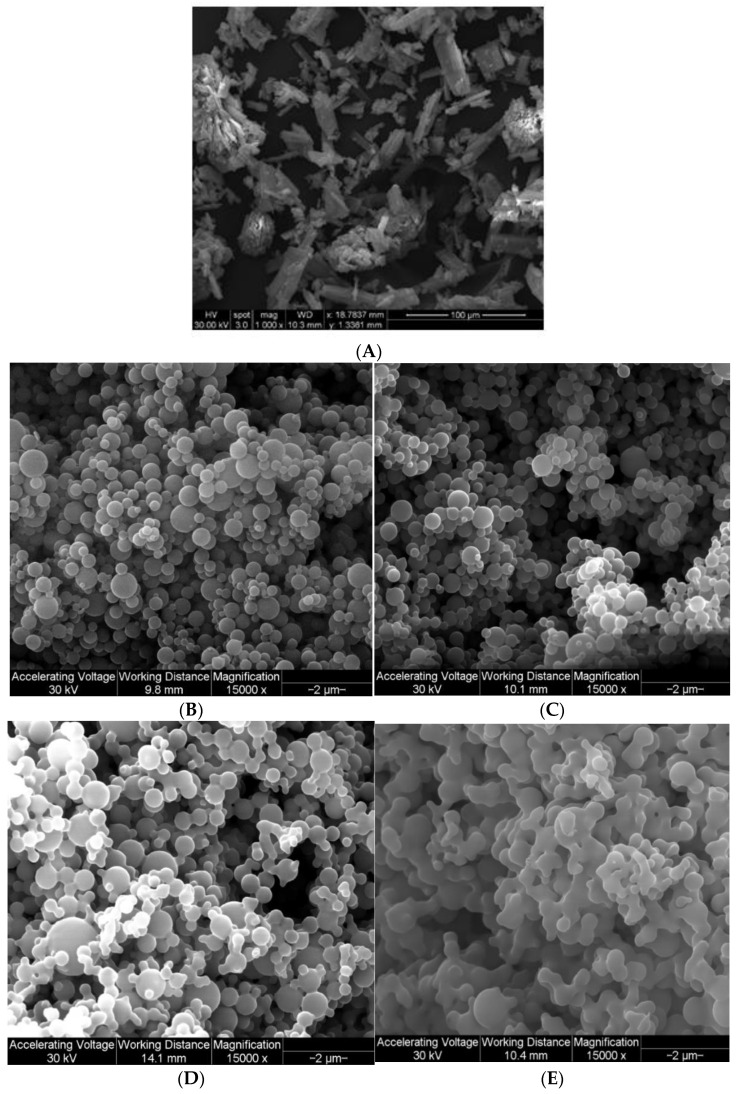
SEM micrographs of: (**A**). raw FAS; (**B**). SD FAS 25% PR; (**C**). SD FAS 50% PR; (**D**). SD FAS 75% PR; and (**E**). SD 100% PR.

Raw Fasudil particles were non-spherical and flat plate-like flakes having a large size and wide size distribution, all of which are particle properties unsuitable for inhalation aerosolization, as shown in Figure 2. Following advanced organic solution spray drying in closed-mode of dilute feed solutions, all pump rates generated small particles with a smooth surface morphology. Particularly, at PR 25% and 50% Fasudil formed small particles which exhibits smooth and spherical morphology. However, at higher pump rates particle morphology deviated from sphericity exhibiting solid bridging between the particles, which was prominently observed with particle SD at 100% PR.

**Figure 3:**
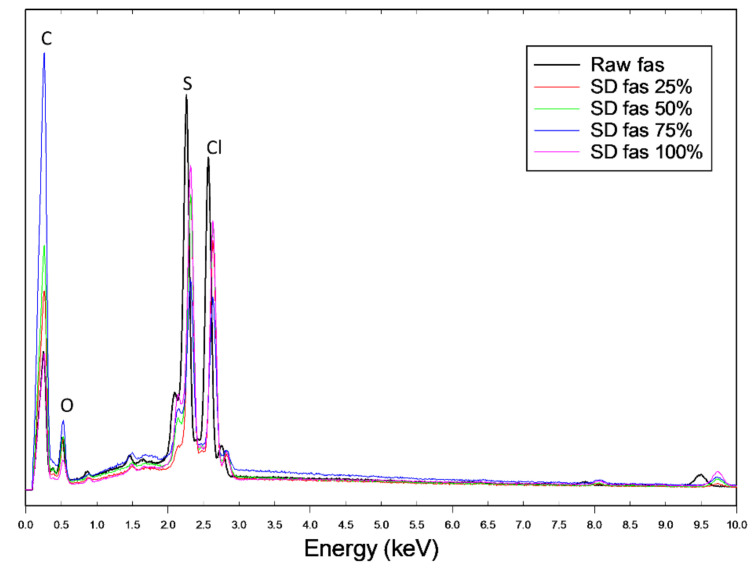
EDX atomic composition spectra of raw FAS and SD FAS showing characteristic peaks for carbon (C), oxygen (O), sulfur (S), and chlorine (Cl) atoms.

The energy dispersive X-ray (EDX) spectra of the powders are shown in Figure 3. For chemical identification of Fas, the characteristic Kα line (peaks) of sulfur (S) is seen at 2.3 keV and the Kα line of chlorine (Cl) is seen at 2.63 keV. The Kα lines of carbon (C) at 0.257 keV and oxygen (O) at 0.526 keV obscure the peaks of nitrogen (N) which is usually seen at 0.392 keV. The peaks corresponding to S and Cl are unique identifying atoms for FAS i.e., not C, H, or O. All SD FAS powders possess these peaks in their EDX spectrum, suggesting that FAS exists as an acid salt before and after spray drying.

### 3.2. Particle Sizing and Size Distribution Using SEM Micrographs

Table 2 lists the particle size range, mean particle size and standard deviation of the SD FAS particles sized from SEM micrographs using SigmaScan Pro 5.0 software (SYSTAT Software, Inc., San Jose, CA, USA). As seen in Table 2, SD FAS particles were predominantly well within the nanometer size. Specifically, 25% pump rate formed particles in the range of 270 nm–1.67 µm, 50% pump rate formed 343 nm–1.94 µm, 75% pump rate formed 346 nm–3.20 µm and 100% pump rate formed 454 nm–2.78 µm.

### 3.3. X-ray Powder Diffraction (XRPD) 

**Figure 4:**
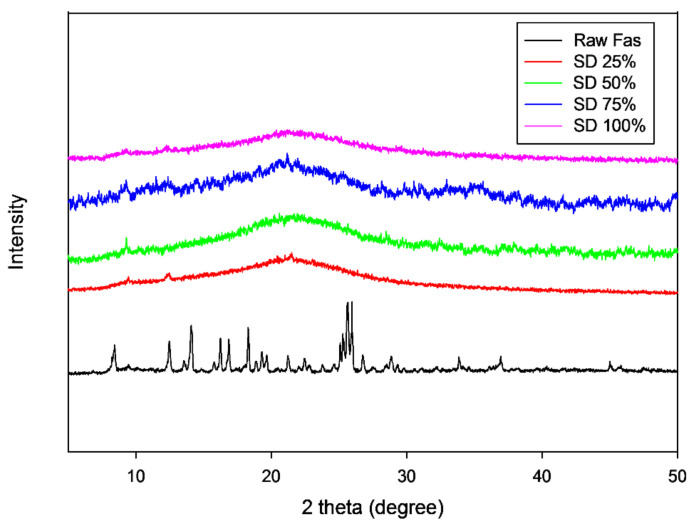
XRPD patterns of raw FAS and SD FAS powders at different spray drying pump rates.

The several diffraction peaks of raw FAS in Figure 4 are suggestive of the presence of long-range molecular order in the raw sample. Raw FAS had distinct and intense X-ray diffraction peaks in the range of 8°–28° 2θ-degrees. The characteristic peaks and other diffraction peaks seen including 8.4°, 14.1°, 16.2°, 16.8°, 18.3°, 19.6°, 21.2°, 22.4°, 25.6° and 25.9° 2θ degree are similar to previously reported X-ray diffraction of Fasudil hemihydrate (40). In contrast, SD FAS particles at all pump rates exhibited no diffraction peaks (Figure 4), unlike the raw drug, which is indicative of a lack of long- range molecular order in SD FAS particles.

### 3.4. Differential Scanning Calorimetry (DSC)

**Figure 5:**
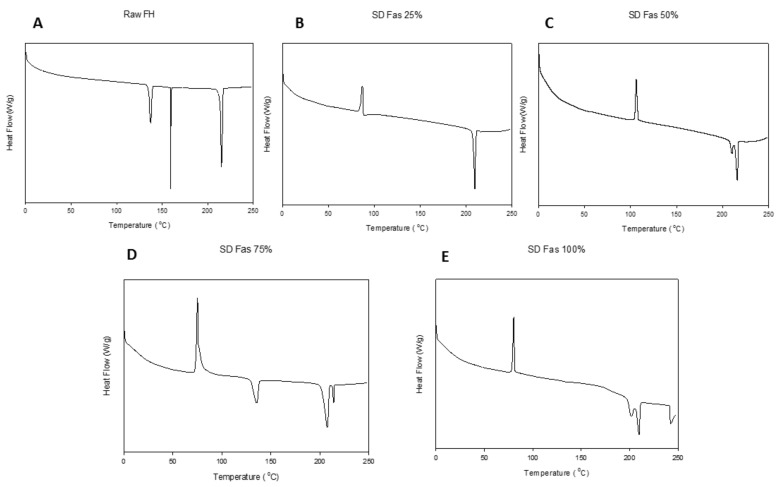
DSC thermograms of Fasudil: (**A**). Raw FAS; (**B**). SD FAS 25% pump rate; (**C**). SD FAS 50% pump rate; (**D**). SD FAS 75% pump rate; and (**E**). SD FAS 100% pump rate. (Exotherm: upward and Endotherm: downward).

As shown in Figure 5, raw Fasudil (a hemihydrate and salt crystalline powder) exhibited three endothermic peaks at 137°, 160° and 215 °C (melting temperature, T_m_). The predicted T_m_ of FAS is around 220 °C (ChemDraw^®^ Ultra Ver. 10.0.; CambridgeSoft, Cambridge, MA, USA).

For all SD FAS powders, a glass transition (T_g_) temperature was evident in the range of 47–52 °C. The SD FAS particles at all pump rates also possessed an exotherm between 75–108 °C indicative of a molecular disorder-to-order phase transition. However, 25% PR exhibited a single endotherm around 209 °C after the transition, while other pump rates exhibited, at least, two endotherms. The exact transition temperatures are listed in Table 3.

### 3.5. Hot-Stage Microscopy (HSM) under Cross-Polarizer Lens

**Figure 6:**
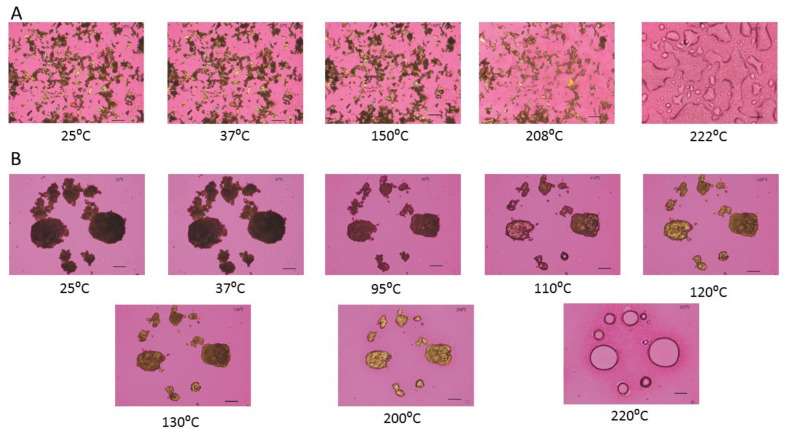

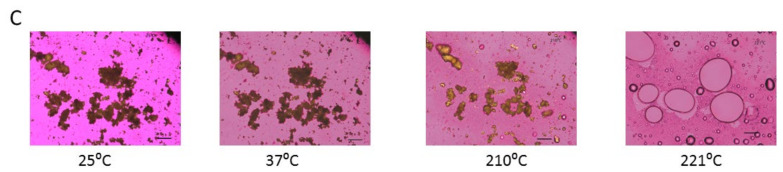
Representative HSM images of: (**A**). Raw FAS; (**B**). SD FAS 25% pump rate; and (**C**). SD FAS 100% pump rate. Scale bar in each image is 10 µm.

As visualized by cross polarized light microscopy, raw Fasudil exhibits no change in birefringence until 150 °C, where there was a change in birefringence followed by the start of melting at 208 °C with a cloudy appearance as shown in Figure 6. The particles exhibited birefringence at room temperature and at all temperatures below T_m_. Birefringence decreased during melting and was absent following melting.

SD FAS (25%) birefringence remained unchanged until 95 °C (Figure 6). After that a change in particle size similar to particle shrinkage was seen followed by the appearance of birefringence around 110 °C, then change in birefringence around 120 ° and 130 °C and finally melting above 200 °C. Similarly, SD FAS 50% PR and SD FAS 75% PR showed particle shrinkage and several changes in birefringence after 93 °C and 43 °C, respectively, before they melted above 200 °C (data not shown). SD FAS at 100% showed no changes in birefringence until 210 °C until it started to melt. 

### 3.6. Karl Fisher Titration (KFT)

The measured residual water content values for raw FAS and SD FAS powders are tabulated in Table 4. As seen in Table 4, raw Fasudil powder had a mean residual water content of 2.97% *w*/*w*. SD FAS nanoparticles/microparticles had mean residual water values in the range of 2.45–3.22% *w*/*w*.

### 3.7. Confocal Raman Microspectroscopy (CRM) and Chemical Imaging

**Figure 7:**
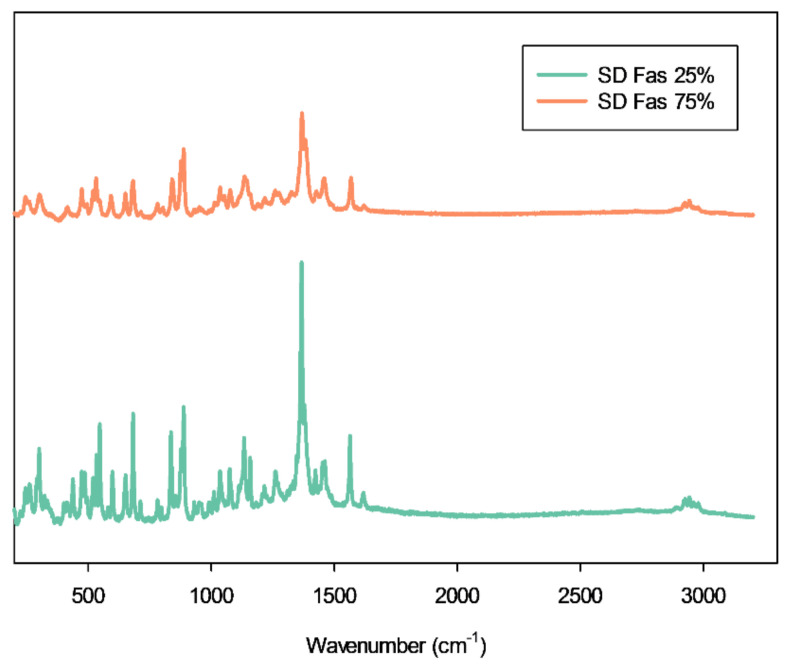
Representative Raman spectra of selected SD FAS formulations obtained using 785 nm laser.

At 785 nm laser the Raman peaks of Raw FAS were seen at Raman shifts of 112 cm^−1^, 841 cm^−1^, 1366 cm^−1^, 1382 cm^−1^, and 1566 cm^−1^. The Raman peaks seen in Figure 7 of representative SD FAS powder shows that the Raman shift didn’t change after spray drying. 

### 3.8. Attenuated Total Reflectance—Fourier Transform Infrared (ATR-FTIR) Spectroscopy

**Figure 8:**
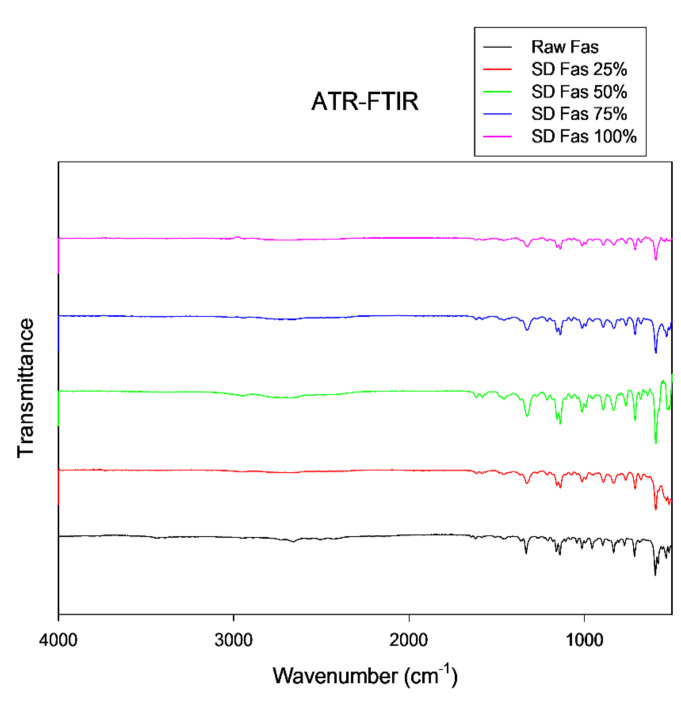
ATR-FTIR spectra of Raw and SD FAS at different spray drying conditions.

Assigning groups to every peak in IR spectrum is difficult since there were many overlapping bands. However, some characteristic peaks of FAS were identified in the ATR-FTIR spectrum in Figure 8 at approximately 1159 cm^−1^ (SO_2_ symmetric stretching) low energy for symmetric stretch and slightly more energy for an anti-symmetric stretch at 1332 cm^−1^ (SO_2_ antisymmetric stretch) characteristics of sulfonamide group. The slight peak seen around 1620 cm^−1^ (stretching of aromatic ring) is indicative of isoquinoline. These are similar to previous reports of FAS hemihydrates [39]. The spectral pattern seen at fingerprint region (<1500 wavenumber) was consistently observed in all SD FAS and raw FAS samples where the peaks were found at ~710 cm^−1^, 836 cm^−1^, 892 cm^−1^, 1012 cm^−1^, 1136 cm^−1^, and 1325 cm^−1^.

### 3.9. In Vitro Aerosol Dispersion Performance

**Figure 9:**
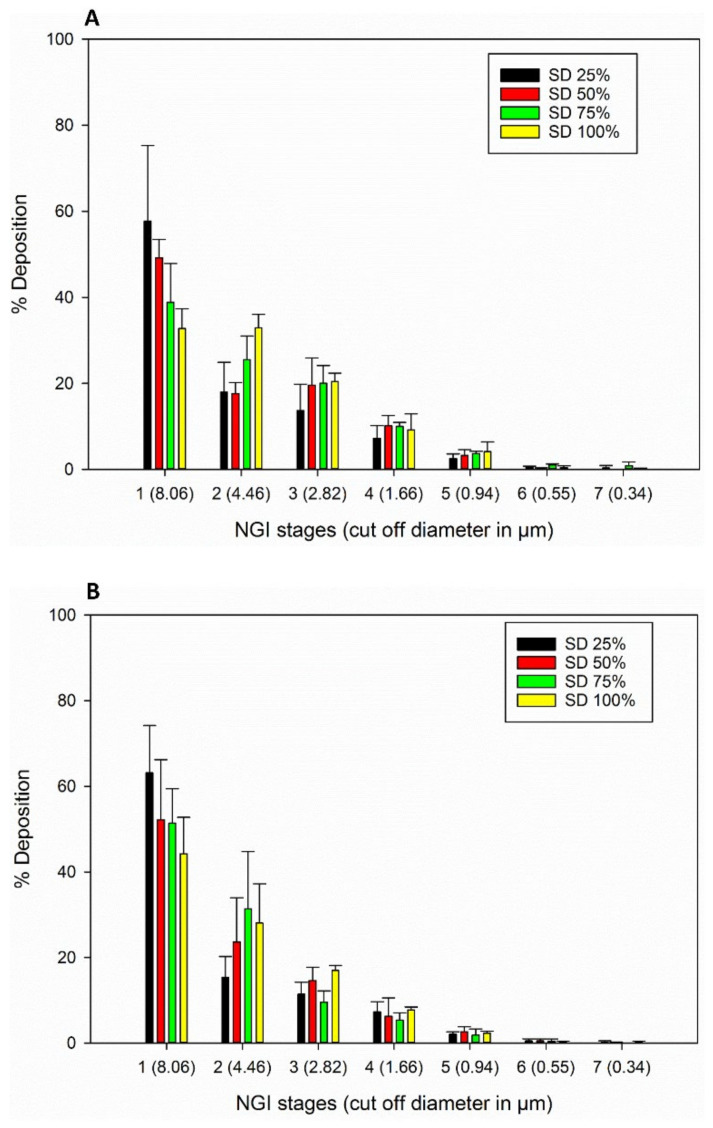

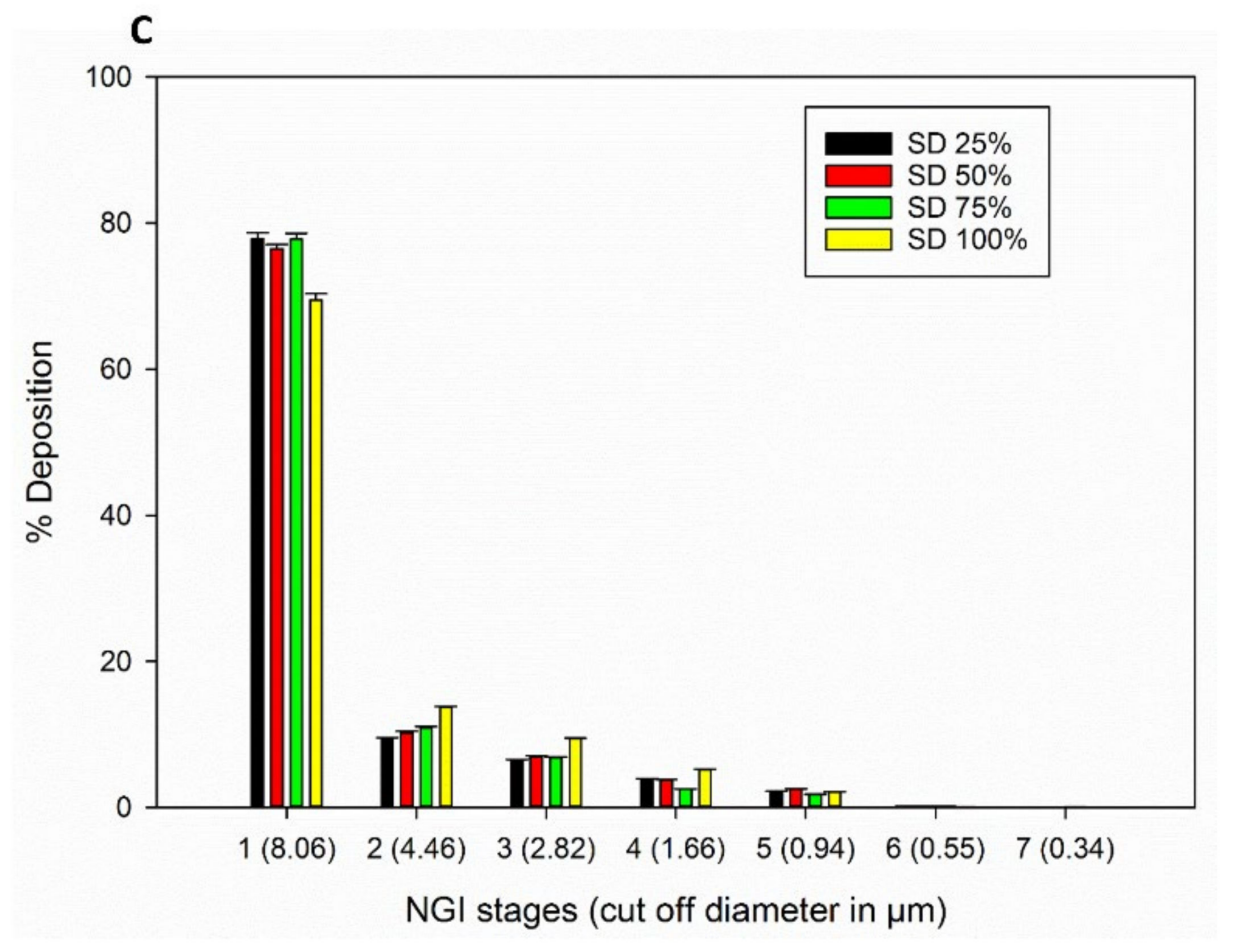
In vitro aerosol dispersion performance of SD FAS powders from all 4 spray drying pump rates using the NGI and the FDA-approved human DPI devices: (**A**). Aerolizer^®^; (**B**). Neohaler^®^; and (**C**). HandiHaler^®^.

The in vitro aerosol NGI stage deposition profiles of the SD FAS particles for all 3 DPI devices are presented in Figure 9. All SD FAS powders aerosolized readily and had measurable aerosol deposition on all NGI stages for all three DPI devices. Aerolizer^®^ and Neohaler^®^ had more deposition on lower stages compared to HandiHaler^®^. The aerosol dispersion performance properties are listed in Table 5. All 3 DPI devices generated high ED, FPF, and RF values for all the SD FAS powders. The Neohaler^®^ DPI device, when used with the SD FAS 75% PR formulation, produced the highest FPF of ~38%, followed by Aerolizer^®^ with 50% SD Fas.

### 3.10. In Vitro Cell Viability

**Figure 10:**
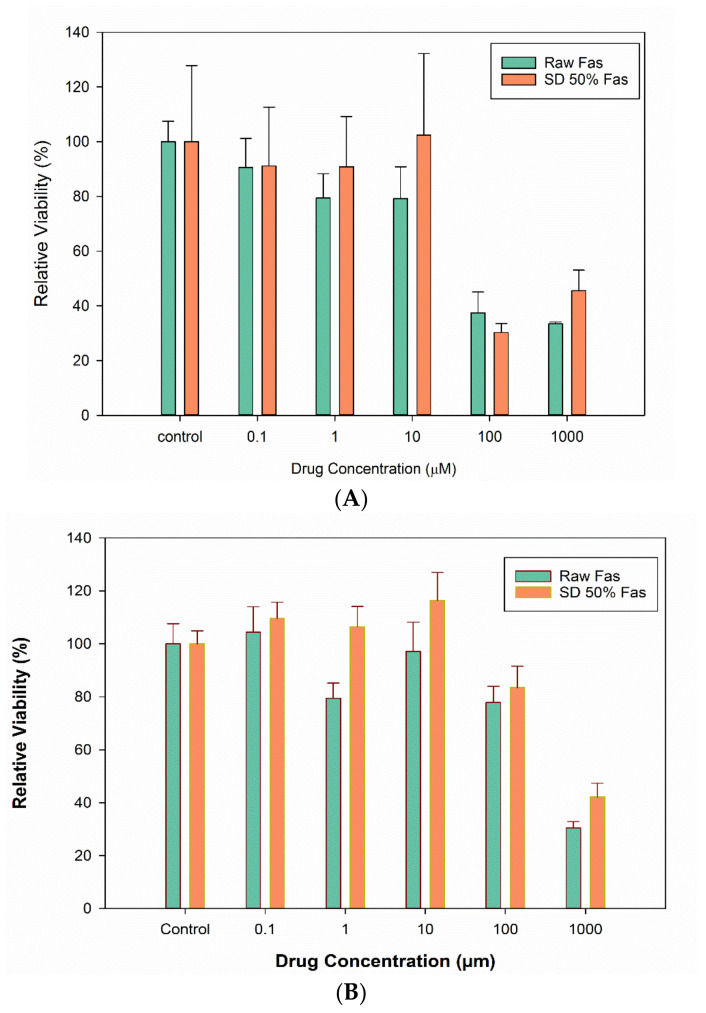
In vitro cell viability of human pulmonary cells exposed to different Fasudil drug concentrations of raw FAS and selected SD FAS formulations: (**A**). A549 and (**B**). H358 pulmonary cell lines (*n* = 6).

In vitro cell viability was performed on two pulmonary cell lines from different lung regions: A549 alveolar epithelial cells and H358 bronchioalveolar human cells. Molar concentrations of 0.1 µM, 1 µM, 10 µM, 100 µM and 1000 µM raw and selected SD FAS powders were tested (Figure 10). Viability of both A549 and H358 cell lines was higher at lower concentrations of Fas. At very high concentrations of 100 µm and 1000 µm, the cell viability was significantly different from that of the control (treated with the media) for H549 cells.

### 3.11. In Vitro Transepithelial Electrical Resistance Analysis

**Figure 11:**
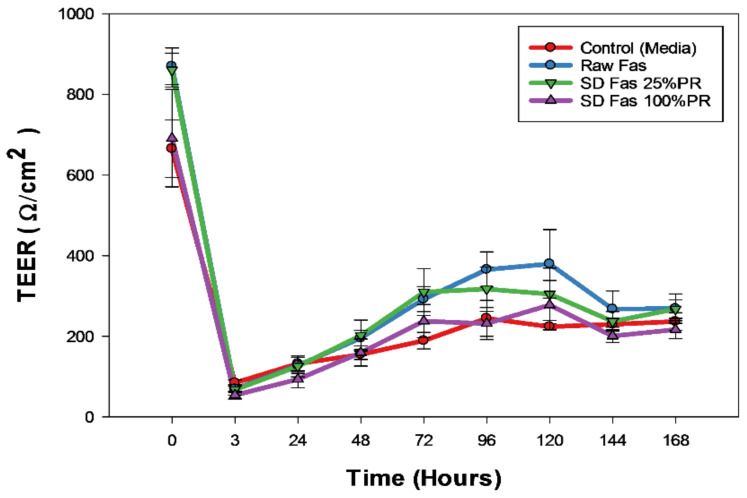
In vitro transepithelial electrical resistance (TEER) analysis of Calu-3 human lung epithelial cells from the large airways exposed to representative aerosolized formulations under air-interface culture (AIC) conditions.

Figure 11 displays the result of TEER measurement of Calu-3 cell line for up to seven days after treating with aerosolized Fasudil (100 µM) of the select SD formulations (low PR and high PR). All tested formulations had an initial reduction in transepithelial electrical resistance within the first 3 h after treatment. After 3 h, the electrical resistance increased steadily in a similar manner to plateau at the same plateau value of the control (aerosolized media).

## 4. Discussion

To our knowledge, this is the first study on solid-state respirable Fasudil nanoparticles/inhalation as DPIs. In addition, this study is the first to report on advanced organic solution spray drying closed-mode under our conditions to rationally design and development Fasudil into solid-state nanoparticles/microparticles with particle solid-state properties tailored and optimized for targeted pulmonary inhalation delivery. This study is the first to report on the interactions of these Fasudil formulations with DPI devices, as tested with three FDA-approved human DPI devices and the influence on aerosol dispersion. Moreover, this study is the first to demonstrate interactions and safety on three different human respiratory cells from different lung regions. 

There have been previous studies reported by our group demonstrating that advanced spray drying in closed-mode using a dilute organic solution of drug feed produce small solid-state particles in the nanometer size range [40,41,42]. As seen from the SEM micrographs (Figure 2), individually spray dried FAS formed small nanoaggregates/nanospheres which have unique advantages for pulmonary drug delivery [43]. The pump rate influenced the size and shape of these particles. An increase in pump rate from 25% (low PR) to 100% (high PR) caused the shape of the particles to be less spherical. At 100% pump rate (high PR), solid sintering between the spray dried particles was observed. Specifically, at the low pump rate of 25% and medium pump rate of 50%, the particles were smaller and spherical compared to the particles at higher pump rates of 75% and 100%. The sphericity of the particles changed as the pump rate increased; this pattern could be due to the difference in the feed rate of the solution, which in turn, affects the residence time of the particles in the primary spray drying chamber. Thus, it appears that the increased residence time allowed for long drying times at the lower feed rates (i.e., lower pump rate) enabled spherical particle formation. The comprehensive solid-state characterization of the powders using thermal analysis and spectroscopic techniques, suggested that spray drying process hasn’t caused chemical degradation of Fasudil molecule. 

Thermal analysis by DSC (Figure 5 and Table 3), diffraction analysis by XRPD (Figure 4), and direct visualization by HSM (Figure 6) are all in good agreement demonstrating that raw Fasudil exists in the crystalline state and SD FAS powders are non-crystalline under the reported advanced spray drying conditions. DSC thermal analysis of raw Fasudil shows three sharp endotherm peaks (T_peaks_). The main phase transition peak characteristic of a solid-to-liquid melting (T_m_), which is a first-order thermodynamic equilibrium phase transition, occurs at 215 °C. Fasudil has been reported to have crystalline polymorphic phases in the solid-state including a hemihydrate and a trihydrate, where the hemihydrate was found to be the stable form. The presence of two endothermic peaks prior to the T_m_ on the DSC thermogram for raw Fasudil is consistent with the presence of solid-state crystalline polymorphs. The peak at 137 °C is possibly dehydration of the hemihydrate followed by melting of the anhydrous form 215 °C.

HSM of raw Fasudil confirms the presence of birefringence at temperatures below T_m_ which is a characteristic hallmark feature of organic crystalline materials. HSM also confirmed that T_m_ of Fasudil occurs above 200 °C as birefringence disappeared and liquid droplets formed. The XRPD diffractograms show sharp peaks at specific angles which is a characteristic hallmark feature of organic crystalline materials.

Contrastingly, the spray-dried Fasudil nanoparticulate/microparticulate powders all exhibited a clear amorphous glass-to-rubber transition (T_g_) prior to the T_m_ in the DSC thermograms, as seen in Figure 5. For all SD FAS powders, T_g_ was evident in the range of 47–52 °C. The SD FAS particles at all pump rates also possessed an exotherm between 75–108 °C indicative of a molecular disorder-to-order phase transition. The XRPD diffractograms (Figure 4) for all the SD FAS formulations show the lack of peaks and the characteristic “halo” of the amorphous state. Visualization of the SD FAS powders by HSM shows a lack of birefringency which is characteristic of non-crystalline organic materials. As listed in Table 3, all T_g_ values are above room and biological temperatures (37 °C). The T_g_ is a second-order kinetic phase transition and is characteristic of the amorphous state which is a metastable state relative to the thermodynamically stable crystalline state. There is a clear increase in the T_g_ values with increasing spray drying pump rate which is indicative that a stronger glass is formed as the spray drying pump rate is increased.

Elemental atomic characterization of raw FAS and SD FAS was measured and confirmed by EDX (Figure 3) where the distinctive peaks corresponding to the S, O, and Cl atoms were seen. ATR-FTIR and Raman molecular fingerprinting spectroscopic analyses (Figure 7 and Figure 8) further supported this by showing the characteristic absorption bands in the fingerprint region. EDX of raw FAS and SD FAS powders indicate that FAS exists as an acid salt before and after spray drying.

An important characteristic for inhalable powders is the residual water content, as it directly influences dry powder aerosol dispersion properties through capillary forces between particles [23,44]. Residual water content in pharmaceutical powders is also important for physicochemical stability. The residual water content KFT values (Table 4) were generally around 3% or less for all spray dried Fasudil powders and raw Fasudil. These are very low values which are well within the acceptable range by the FDA for DPIs which more than 6% *w*/*w* is not.

The formulation-device interaction is critically important in DPIs, as it influences aerosol dispersion. As seen in Figure 9 and Table 5, all SD FAS nanoparticulate/microparticulate formulations aerosolized efficiently with all 3 DPI devices. Deposition was measurable on all of the NGI stages. The deposition of the advanced spray dried powders on the NGI stages changed with different FDA-approved human DPI unit-dose capsule devices that vary in shear stress and resistance levels. The Aerolizer^®^ DPI device is a low-shear stress device, while the Neohaler^®^ and the HandiHaler^®^ DPI devices are medium- and high-shear stress devices, respectively. The lower resistance device Aerolizer^®^ generated aerosols with decreased deposition on NGI Stage 1 and increased the mass deposition on NGI Stages 2–7 compared to HandiHaler^®^. Deposition of aerosols generated by the Neohaler^®^ was relatively similar to that of Aerolizer^®^. NGI stages 5–7 have aerodynamic cut-off sizes in the nm size range and NGI Stages 1–4 have aerodynamic cut-off sizes in the µm size range. ED values were very high for all SD FAS aerosols generated by all 3 devices with the Neohaler^®^ resulting in the highest ED values. The FPF values for SD FAS 25% PR aerosols were comparable for all 3 DPI devices. The FPF values for SD FAS 50% PR aerosols were higher when generated by the Neohaler^®^ and Aerolizer^®^ DPI devices than by the HandiHaler^®^. The FPF values for SD FAS 75% PR aerosols were highest with the Neohaler^®^ DPI device, second highest with the Aerolizer^®^ DPI device, and third highest with the HandiHaler^®^ DPI device. The FPF value for SD FAS 100% PR aerosols was highest with the Neohaler^®^ DPI device, second highest with the HandiHaler^®^ DPI device, and third highest with the Aerolizer^®^ DPI device.

**Figure 12:**
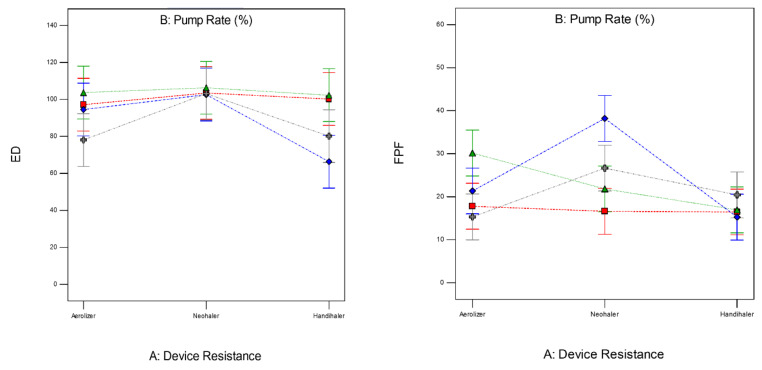

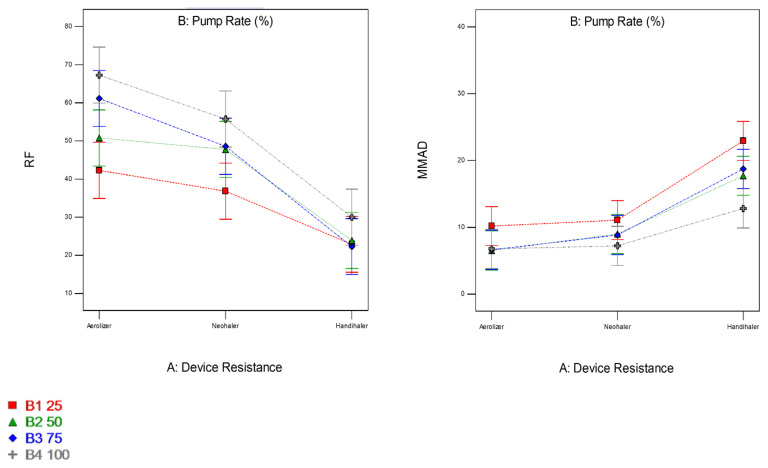
Design of experiments (DOEs) interaction plots (Design Expert^®^ 8.0.7.1 software, Stat-Ease Corporation, Minneapolis, MN, USA) of spray drying pump rate and inhaler device resistance on in vitro aerosol performance parameters for all 4 SD FAS dry powders (25% PR, 50% PR, 75% PR, and 100% PR).

**Figure 13:**
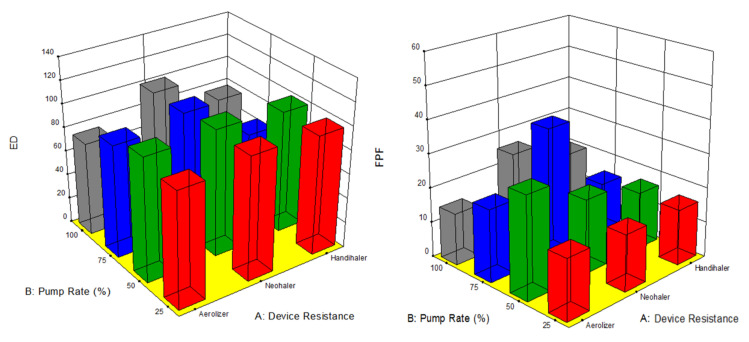

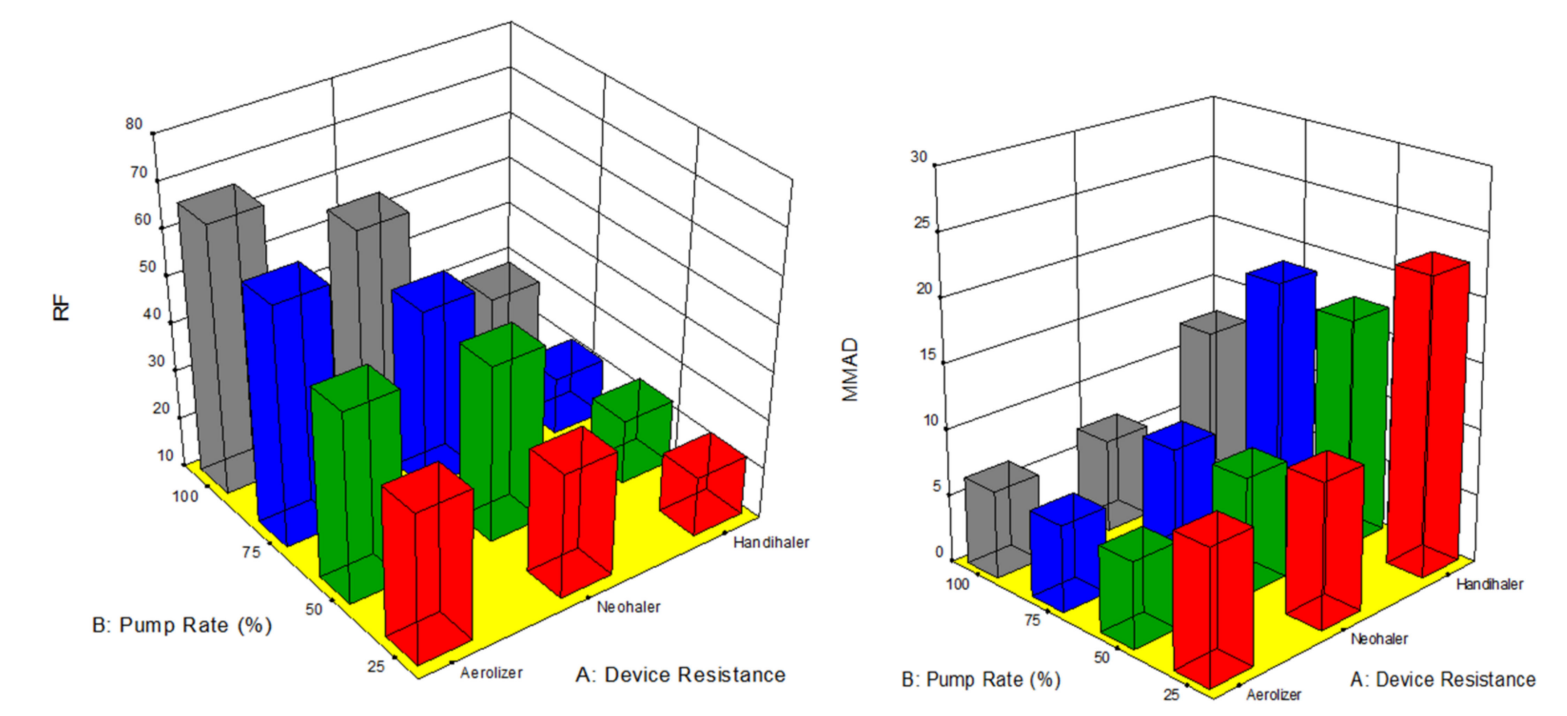
Design of experiments (DOEs) 3-D surface plots (Design Expert^®^ 8.0.7.1 software, Stat-Ease Corporation, Minneapolis, MN, USA) of spray drying pump rate and inhaler device resistance on in vitro aerosol performance parameters for all 4 SD FAS dry powders (25% PR, 50% PR, 75% PR, and 100% PR).

The factors interaction plots shown in Figure 12 and surface 3-D plots shown in Figure 13 reveal the interaction of spray drying pump rate and the DPI device shear stress factors on influencing aerosol dispersion of SD FAS particles. All of the SD FAS nanoparticulate/microparticulate powders produced at different spray drying pump rates had the same or lower FPF values when aerosolized using the HandiHaler^®^ DPI device. However, the FPF value is increased for some pump rates with low resistance device while for the other pump rates with medium resistance device. This explains that the de-aggregation of the particles depends on the combination of formulation and the device properties. The RF of all the SD FAS particles decreased with high resistance inhaler, suggesting insufficient detachment of the primary particles. One-way ANOVA statistical analyses indicated that the device resistance and pump rate had statistically significant effects on the aerosol dispersion performance parameters. This predictive model serves as a pre-screening tool to rationally select and optimally match of the SD FAS formulation with a human DPI device to achieve superior aerosol performance. For example, 75% SD FAS performs better with Neohaler^®^, while 25% SD FAS performs well with Aerolizer^®^.

At the sizes that are presented in this study which are inhalable nanometer/small micrometer sizes, the surface forces of solid-state particles are significant and hence have increased interparticulate interactions by virtue of high surface area. Particle morphology and surface morphology also influence aerosol dispersion of powders as DPIs. Elongated shaped particles will have more contact area and increased interparticulate interactions (i.e., van der Waals, electrostatic and capillary forces) leading to more aggregation and poor aerosol dispersion [45,46,47]. The deaggregation of DPI formulation also depends on the airflow pattern and shear stress of the DPI device [44,48,49,50,51]. Some strategies to improve aerosol efficiency include modifying the physicochemical properties such as size, shape, morphology, decreased surface energy, or blending with a carrier that can decrease the drug-drug cohesive energy [44]. Powder dispersibility is also improved in particles with a certain degree of corrugation due to reduced cohesion forces [52] as opposed to particles with completely smooth surface. In the current study, all SD particles had smooth surface and exist as nanoaggregates, where the aerosol performance was influenced by particle shape and dispersibility [53,54]. DPI aerosol performance is also directly influenced by surface amorphous content leading to enhanced water vapor sorption and capillary forces, surface hydrophobicity, and electrostatics [45,46].

The in vitro human respiratory cell experiments (Figure 10 and Figure 11) on A549 and H358 cells from different lung regions show that the raw Fasudil drug and SD FAS formulations doesn’t significantly affect cell viability of these human pulmonary cell lines over a wide dose range studied. Raw Fasudil and SD FAS powders exhibit similar safety profiles at the concentrations used on both human lung cell types. On A549 human alveolar lung cells, Fasudil at very high concentrations of 100 µM and 1,000 µM, a significant decrease in viability was observed. On H358 human bronchioalveolar lung cells, safety was achieved at all of the Fasudil concentrations studied except for the very high concentration of 1000 µM. Typical therapeutic aerosols achieve efficacy at much lower concentrations. After 3 days, the TEER values of large airway lung cells (CaLu-3) under AIC conditions reached their plateau recovery potential which was the same as the control suggesting membrane recovery following exposure to aerosolized Fasudil. This recovery trend for CaLu-3 human lung cells is similar to recovery observed under AIC conditions with other aerosolized drugs reported by us previously [34,37,38]. The in vitro cell viability and in vitro TEER at the AIC demonstrate the safety profile for raw Fasudil and SD FAS formulations.

## 5. Conclusions

To our knowledge, this is the first study to report on solid-state respirable Fasudil nanoparticles/microparticles as DPIs and with comprehensive physicochemical characterization following successful particle engineering design by advanced organic solution spray drying closed-mode. The solid-state nanoparticles/microparticles had solid-state properties that were tailored and optimized for targeted pulmonary inhalation delivery. This study is the first to report on the interactions of these Fasudil formulations with DPI devices, as systematically tested with three FDA-approved human DPI devices and the influence on aerosol dispersion. Interactions and safety on three different human respiratory cells from different lung regions were demonstrated in vitro. These innovative Fasudil inhalable powders have the potential to be a new “first-in-class” drug treatment of several pulmonary diseases such as PH, asthma, and fibrosis.

## Figures and Tables

**Table 1:** Spray drying parameters for advanced spray-dried (SD) Fasudil powders at a feed concentration of 0.5% *w*/*v* from methanol (MeOH) solution in closed-mode.

Feed Pump Rate (PR) %	25% (7.5 mL/min)	50% (15 mL/min)	75% (22.5 mL/min)	100% (30 mL/min)
Inlet Temperature (°C)	149–152	150–151	150–151	150–152
Outlet Temperature (°C)	76–88	67–68	55–65	43–56
Aspirator rate (m^3^/h)	37.5	37.5	37.5	37.5
Atomization gas flow rate (L/h)	670	670	670	670

**Table 2:** Particle sizing using image analysis on SEM micrographs (*n* ≥ 100 particles).

Spray Drying Pump Rate (%)	Size Range (µm)	D_50_ (Mean) Size (µm)
25	0.27–1.677	0.068 ± 0.023
50	0.343–1.938	0.855 ± 0.302
75	0.346–3.199	1.075 ± 0.510
100	0.454–2.778	1.168 ± 0.507

**Table 3:** DSC thermal analysis. (*n* = 3, mean ± standard deviation).

Powder Identification	Spray Drying Pump Rate (%)	DSC Peaks	T_peaks_ (°C)	ΔH (W/g)	T_g (peak)_ (°C)	ΔC_p_ (J/g °C)
Raw FAS	N/A	Transition 1 Transition 2 T_m_ (Fas)	137.19 ± 0.19 159.86 ± 4.94 215.01 ± 0.30	55.06 ± 3.29 16.93 ± 3.62 105.5 ± 2.89	N/A	N/A
SD FAS	25	T_c_ T_m_ (Fas)	86.10 ± 1.91 209.28 ± 0.71	45.35 ± 3.09 88.18 ± 12.69	46.91 ± 1.05	1.33 ± 0.17
SD FAS	50	T_c_ T_m_ (Fas) Tm (Fas)	106.66 ± 0.79 209.47 ± 0.37 215.79± 0.36	41.54 ± 11.58 8.54 ± 2.04 44.26 ± 14.19	47.07 ± 0.44	1.15 ± 0.44
SD FAS	75	T_c_ T_m_ (Fas) T_m_ (Fas)	108.13 ± 0.99 210.52 ± 0.54 216.61 ± 0.42	41.50 ± 5.76 45.81 ± 7.8 23.59 ± 1.60	49.47 ± 3.42	0.49 ± 0.17
SD FAS	100	T_c_ T_m_ (Fas) T_m_ (Fas)	76.25 ± 3.26 186.84 ± 20.81 211.51 ± 2.07	39.13 ± 8.46 16.66 ± 0.22 34.48 ± 5.49	51.80 ± 1.34	0.50 ± 0.22

**Table 4:** Residual water content quantified by KFT. (*n* = 3, mean ± standard deviation).

Powder Identification	Spray Drying Pump Rate (%)	Residual Water Content (% *w*/*w*)
Raw FAS	N/A	2.97 ± 0.06
SD FAS	25	3.22 ± 0.56
SD FAS	50	2.57 ± 0.36
SD FAS	75	2.45 ± 0.49
SD FAS	100	2.73 ± 0.57

**Table 5:** In vitro aerosol dispersion performance properties of SD FAS powders at four different pump rates using the FDA-approved human DPI devices, Aerolizer^®^, Neohaler^®^ and HandiHaler ^®^.

	Aerolizer^®^	Neohaler^®^	HandiHaler^®^
	SD FAS 25%		
FPF (%)	17.79 ± 3.78	16.63 ± 4.78	16.65 ± 1.76
RF (%)	42.28 ± 22.14	36.82 ± 17.75	22.17 ± 2.67
ED (%)	97.16 ± 2.86	103.49 ± 1.26	98.56 ± 2.49
MMAD (µm)	10.18 ± 5.26	11.07 ± 5.25	22.33 ± 7.06
GSD	2.38 ± 0.36	2.78 ± 0.26	3.58 ± 0.57
	**SD FAS 50%**		
FPF (%)	30.16 ± 3.74	21.83 ± 5.94	16.53 ± 3.06
RF (%)	50.79 ± 22.19	47.78 ± 22.94	23.36 ± 2.15
ED (%)	103.71 ± 2.76	106.29 ± 3.17	99.58 ± 0.44
MMAD (µm)	6.53 ± 2.87	9.00 ± 4.49	17.83 ± 1.60
GSD	2.2 ± 0.22	2.37 ± 0.38	3.27 ± 0.05
	**SD FAS 75%**		
FPF (%)	21.35 ± 7.05	38.21 ± 10.42	15.2 ± 0.59
RF (%)	61.15 ± 27.24	48.59 ± 21.79	20.07 ± 4.05
ED (%)	94.52 ± 10.42	102.59 ± 1.52	92.64 ± 6.62
MMAD (µm)	6.62 ± 2.98	8.84 ± 3.86	18.77 ± 0.44
GSD	2.38 ± 0.31	2.12 ± 0.477	3.15 ± 0.20
	**SD FAS 100%**		
FPF (%)	15.32 ± 1.44	26.65 ± 7.83	21.082 ± 1.40
RF (%)	67.27 ± 29.31	55.74 ± 24.88	30.83 ± 2.23
ED (%)	78.07 ± 1.52	103.12 ± 0.98	80.57 ± 8.97
MMAD (µm)	6.77 ± 3.11	7.22 ± 3.15	12.54 ± 1.11
GSD	1.86 ± 0.33	2.15 ± 0.18	3.04 ± 0.41

*n* = 3, mean ± standard deviation.

## Data Availability

The data presented in this study are available on request from the Corresponding Author.
